# Combining systems thinking approaches and implementation science constructs within community-based prevention: a systematic review

**DOI:** 10.1186/s12961-023-01023-4

**Published:** 2023-08-28

**Authors:** Jillian Whelan, Penny Fraser, Kristy A. Bolton, Penelope Love, Claudia Strugnell, Tara Boelsen-Robinson, Miranda R. Blake, Erik Martin, Steven Allender, Colin Bell

**Affiliations:** 1 School of Medicine, Deakin University, Geelong, Australia; 2 School of Health and Social Development, Deakin University, Geelong, Australia; 3 School of Exercise and Nutrition Sciences, Deakin University, Geelong, Australia; 4 Institute for Health Transformation, Geelong, Australia; 5 Global Centre for Preventive Health and Nutrition, Geelong, Australia; 6 Institute for Physical Activity and Nutrition, Geelong, Australia

**Keywords:** Implementation science, System science, Complexity, Methodology

## Abstract

**Background:**

Systems science offers methods for designing population health interventions while implementation science provides specific guidance for successful implementation. Integrating systems and implementation science may strengthen implementation and enhance and sustain systemic change to achieve system-level outcomes. Little is known about the extent to which these two approaches have been integrated to date. This review aimed to identify and synthesise the peer-reviewed literature that has reported the combined use of systems thinking approaches and implementation science constructs (within the same study), to deliver population health interventions.

**Methods:**

A systematic literature search of peer-reviewed original research was conducted across six databases from 2009 to 2021. Journal manuscripts were included if they: (1) reported on a population health study conducted in a community, (2) reported the use of a systems method in the design of the intervention, and (3) used an implementation science theory, framework or model in the delivery of the intervention. Data extracted related to the specific systems methods and definitions and implementation science constructs used. The Mixed Methods Appraisal Tool (MMAT) was used to assess study quality.

**Results:**

Of the 9086 manuscripts returned, 320 manuscripts were included for full-text review. Of these, 17 manuscripts that reported on 14 studies were included in the final extraction. The most frequently reported systems methods were a ‘whole of community systems approach’ (*n* = 4/14) and ‘community-based system dynamics’ (*n* = 2/14). Nineteen different implementation science theories, frameworks and models were used for intervention delivery, with RE-AIM being the only framework used in more than one study.

**Conclusion:**

There are few published peer-reviewed studies using systems thinking and implementation science for designing and delivering population health interventions. An exploration of synergies is worthwhile to operationalise alignment and improve implementation of systems thinking approaches.

*Review protocol registration* PROSPERO CRD42021250419.

**Supplementary Information:**

The online version contains supplementary material available at 10.1186/s12961-023-01023-4.

## Contribution to the literature

- Research has called for the adoption of systems approaches to tackle complex problems, however there is limited understanding of ‘how to’ implement solutions designed using systems science.
- Multiple studies have reported the use of systems methods in the design of interventions, but few have yet incorporated implementation science into the delivery of the intervention.
- Although we found some scientific evidence of interventions combining both implementation science and systems science, there was no clear guidance on the role implementation science could play and how these two sciences can best be utilised together.
- These findings contribute to recognized gaps in the literature, including the potential of implementation science to contribute significantly to the implementation of systems approaches to addressing complex problems.

## Background

Non-communicable diseases (NCDs) are responsible for almost 70% of all deaths worldwide [1]. Many of these deaths are preventable if causal modifiable risk factors, such as tobacco use, physical inactivity and unhealthy diets were addressed [2]. Relationships between drivers for NCDs (e.g. individual, environmental, societal, political etc.) are complex and dynamic, leading to calls for the adoption of a systems thinking approach [3, 4]. Systems thinking approaches extend socioecological model (SEM) approaches by promoting work across multiple levels of the SEM and actively engaging feedback loops, time delays and seeking effective intervention points [5].

Systems science is a broad field of study that incorporates methodologies with a common goal of understanding complexity [6]. Properties of complex systems include emergence, which is the collective behaviours of the system, outcomes that would not be produced by individual components alone [7]. Complex systems are also adaptive and change behaviour in response to their current environment, therefore changes to a system are likely to create further adaptive responses from within the system [8]. Systems utilise feedback, this circular causality leads to multiple elements within a system impacting others in a circular way, causing self-reinforcing or self-correcting system behaviours [8]. Therefore when working with systems, an emphasis is placed on the ‘whole’ and the importance interactions between components is observed and managed where possible [9, 10]. Many systems methods (e.g. stock and flow diagram, causal loop diagram, systems dynamics modelling etc.) [6], facilitate this enhanced understanding of systems components and their interrelationships, and some methods also assist in the identification of solutions [11] and where resources are best placed to facilitate systems change [12–14].

Systems thinking approaches are increasingly being used in population health, particularly in community health and wellbeing [10, 15]. These efforts have targeted outcomes including determinants of obesity [16], initiatives related to school health [17], fruit and vegetable intake in children [18], policy options for tobacco control [19], and mental health [20]. There are over 25 different systems methods that may be relevant to population health interventions [21, 22]. A recent review of system dynamics and agent-based models describes the expansion of modelling into population health over the last 10 years [23], but only four of these models mentioned the term ‘implementation’, and, in each case, this related to policy implementation [23]. There is emerging recognition of the potential of systems-oriented implementation research [24].

Implementation science, as the study of methods to promote the systematic uptake of research into practice [27], has potential to provide guidance to the implementation of systems approaches [25, 28], through theoretical constructs that provide into successful implementation [27]. Historically, an implementation science ‘evidence to practice’ journey has been depicted as a linear process; however this does not align with the theory and practice of systems approaches to address complex problems [29]. To enhance the alignment between implementation science and systems science, the dynamic properties inherent within complex systems need to be considered [29]. Some implementation science theories, frameworks and models (TFMs) may more easily facilitate such adaptation. Overall TFMs have three broad aims: to describe and/or guide the process of translating research into practice; to understand the determinants that influence implementation; and to evaluate implementation outcomes [27]. A systems intervention, like any intervention, may require one or more types of these TFMs to guide the evidence to full implementation.

Within implementation science, over 150 TFMs exist, with less than a quarter being utilised within ‘system’ change interventions [28]. Historically, an implementation science ‘evidence to practice’ journey has been depicted as a linear process, however this does not align with the theory and practice of systems approaches to address complex problems [29]. To enhance the alignment between implementation science and systems science, the dynamic properties inherent within complex systems need to be considered [29]. The potential for combining the two sciences of implementation and systems has previously been identified [30]. Northridge [30] sought to enhance implementation science through the addition of best principles from systems science, e.g. problem modelling, important elements vs quantifiable elements, boundaries and a multi-component approach. Some TFMs may more easily facilitate such adaptation. Overall TFMs have three broad aims: to describe and/or guide the process of translating research into practice (process frameworks); to understand the determinants that influence implementation (determinants frameworks); and to evaluate implementation outcomes (evaluation frameworks) [26]. A systems intervention, like any intervention, may require one or more types of frameworks to guide the evidence to full implementation.

Interventions have reported a disconnect between systems thinking and implementation science in practice. Gerritsen reported on the use of Group Model Building (a method within community-based system dynamics) [31] to promote fruit and vegetable consumption in a multi-cultural low-income community in West Auckland, New Zealand. GMB helped the community identify community-led (e.g., bottom up) actions for implementation, but did not report on how that implementation would occur [18]. Gerritsen et al. have reported that more work needs to be done to ensure that the implementation aspects are given due consideration in the design phase of GMB research [32]. This difficulty in implementing interventions designed using various systems methods has been echoed by several authors [33, 34]. The Lancet Commission on Obesity noted that poor implementation limits the effectiveness of community interventions, ‘and a greater application of implementation science might help overcome these barriers’ [4].

This review aimed to identify and synthesise the peer-reviewed literature that has reported the combined use of systems thinking approaches and implementation science constructs within the same study, to deliver population health interventions. The purpose was to inform enhanced intervention planning and research through combining the strengths of both implementation and systems science together in real-world applications. This review asks: What systems thinking and implementation science approaches have been used in combination to deliver population health interventions?

## Methods

This review was prospectively registered with PROSPERO (CRD42021250419) and follows the Preferred Reporting Items for Systematic Reviews and Meta-Analysis (PRISMA) guidelines [35].

### Search strategy

A systematic search was conducted of the online databases of Ovid Medline, Embase, Cochrane Central; and within the EBSCO Host platform: ERIC, PsycInfo and CINAHL for peer-reviewed studies published in English. The search included articles from 1 January 2009 to date of search–15 March 2021. The year 2009 was chosen as the starting point to align with the initial work of the intervention-level framework [36], the first published attempt to operationalise the systems science work by Donella Meadows on places to act in a system for public health interventions [13]. A research librarian assisted with the development, testing and subsequent translation of the search terms across the multiple databases used. Search terms explored concepts of: health promotion, obesity, population health, community AND systems science or complexity terms AND implementation science terminology AND frameworks, models or approaches. The full search terms can be found in Additional file 1.

Studies were included in this review if they were published in English, and the:

1. study reported on primary or secondary prevention specifically related to the uptake, adoption or implementation of a health promotion intervention, innovation or initiative or evidence-based practice, process, policy (hereafter ‘intervention’) related to healthy eating, physical activity, tobacco control, alcohol and other drugs, or mental health; and
2. intervention took place in one or more community settings or whole of community: e.g. school, workplace, sports club, community health or other; and
3. manuscript explicitly stated that the study used a systems thinking approach; and
4. manuscript described the implementation of an intervention either through an explicit implementation science framework, model or theory; or via an author’s own implementation plan or theory.

In the reporting of results, the terms ‘intervention’, ‘innovation’, ‘initiative’ or ‘evidence-based practice’ are collectively referred to as ‘interventions’.

Studies were excluded if the study:

1. related to ‘treatment’ of a pre-existing medical condition that is not generally considered preventable; or
2. was conducted in controlled settings, e.g., prisons or hospitals.

### Study selection

All titles and abstracts were screened twice. One author (JW) screened all titles and abstracts. The second screening was shared between co-authors (PF, KB, TBR, EM, PL, CS, CB) and discrepancies on inclusion were resolved by CB. All full text articles were screened twice. JW screened all full texts and the second screening was shared between co-authors (PF, KB, EM, PL, CS). Conflicts were resolved by discussion with the two reviewers. Where agreement was not reached (*n* = 2), CB resolved outstanding conflicts.

### Data extraction

Data extraction on the remaining 14 studies (17 manuscripts) was conducted by one author (JW) with the second review shared between co-authors (MB, TBR, KB, PF, PL, CS). PF and JW conducted consensus where disagreement arose between JW and the second data extractor.

The data extraction template collated data on the use of systems science in the design of the study and on the use of implementation science in the implementation of the study as reported by the author of each manuscript. Data extracted included the public health issue addressed, the use of systems terminology, definitions and methods used, study design, and specific implementation science TFMs applied. (Additional file 2).

### Quality appraisal

The Mixed Methods Assessment Tool (MMAT) [37] was applied to all included manuscripts by two co-authors (JW (all), PF, MB, KB, CS, PL, TBR). MMAT was deemed suitable due to its versatility in appraising varying study designs, including qualitative, quantitative and mixed methods. All included studies met the two screening questions of MMAT which are: 1. Are there clear research questions? and 2. Do the collected data allow to address the research questions?, and then were appraised according to study design criteria. Unlike other quality appraisal tools, the MMAT discourages the use of an overall score (Additional file 3).

## Results

**Fig. 1:**
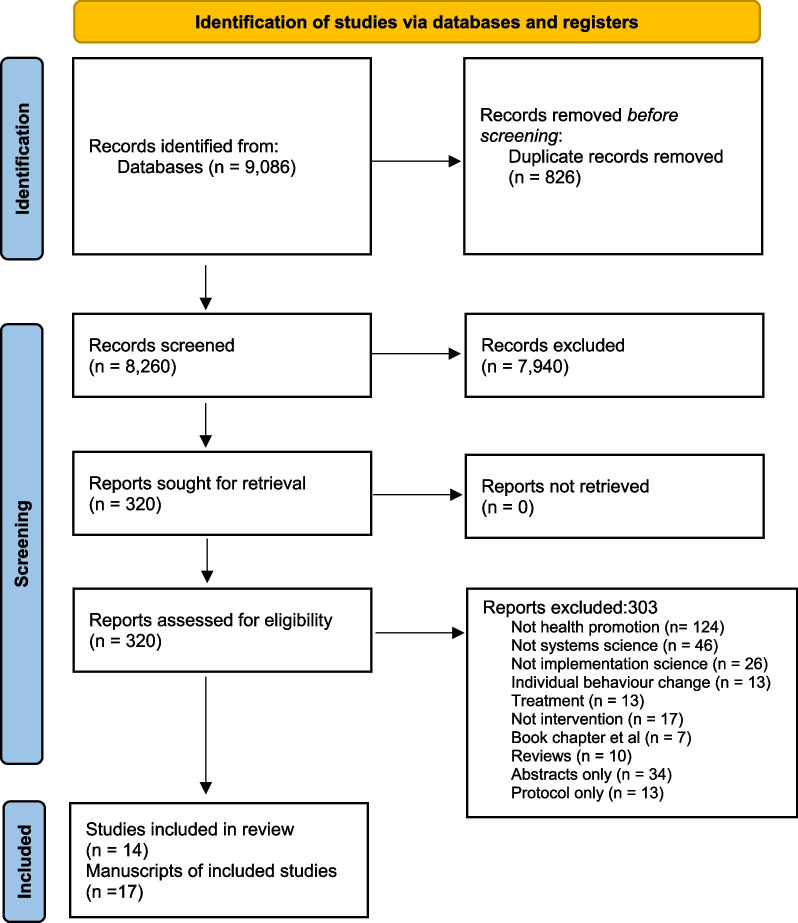
PRISMA diagram

The search returned 9086 manuscripts, of which 826 were duplicates (Fig. 1). Of the remaining 8260 manuscripts, 7940 were excluded based on inclusion/exclusion criteria. Full-text review of the remaining 320 manuscripts meant 303 were excluded, leaving 17 manuscripts. The 17 manuscripts reported on 14 studies. Three studies were described in both a design manuscript and an implementation manuscript which were combined to ascertain the systems and implementation science methods utilised in the studies [38].

Of 14 included studies, three studies were conducted in Australia, four studies in the United States, three studies in the United Kingdom, and one study each of Canada, Austria, Ghana and Mexico, and New Zealand. Of the included studies, 30% reported an intervention duration of two years or less, 50% reported between three- and five-years duration, and 20% reported on studies that lasted six years or more. Thirty percent (30%) of studies aimed to promote health in schools, 30% to prevent childhood obesity, 20% to prevent chronic diseases, and others aimed to promote breastfeeding and food security, general community health and adaptation of evidence-based health programs. Most studies (60%) targeted children (0 to 18 years), 20% targeted all ages, and one study targeted mothers who wished to breastfeed. In terms of study design, 65% utilised mixed methods and 35% were qualitative studies.

Table 1 provides a summary of the studies included in this review, the first author, title, date of publication, country within which the study was conducted, intervention duration, issue addressed, target population and study design.

**Table 1:** Included studies: author, title, country, intervention duration, health issue addressed, target population, and study design

	Study ID	Title	Country	Duration of intervention	Population Health Issue Addressed	Target population	Study design
1	Allender et al., 2019 [39]	Translating systems thinking into practice for community action on childhood obesity	Australia	2016–2017 (1.5 years)	Childhood obesity prevention	Children	Qualitative study reporting on a stepped-wedge cluster randomised controlled trial
2	Amed et al., 2016 [40] *Amed and Kennedy are related studies: SCOPE*	Wayfinding the live 5–2-1–0 initiative—At the intersection between systems thinking and community-based childhood obesity prevention	Canada	2009–2014 (6 years)	Childhood obesity prevention	Children	Mixed methods
3	Kennedy et al. 2019 [41] *Amed and Kennedy are related studies: SCOPE*	Propagating Change: Using RE-FRAME to Scale and Sustain A Community-Based Childhood Obesity Prevention Initiative		2009–2016 (8 years)	Childhood obesity prevention		Mixed methods
4	Ballard et al., 2020 [17] *Ballard and Purnell are related studies*	Community-Based System Dynamics for Mobilizing Communities to Advance School Health	United States	2018–2019 (2 years)	(Complexity of) School health	School students (K-12 education context)	Mixed methods Case study
5	Purnell et al., 2020 [42] *Ballard and Purnell are related studies*	Research to Translation: The Healthy Schools Toolkit and New Approaches to the Whole School, Whole Community, Whole Child Model		2017–2019 [3 years]	Whole health in schools		Mixed methods
6	Bensberg 2021a [43] *Bensberg 2021a and 2021b are related studies: Healthy Together Victoria*	Developing a Systems Mindset in Community-Based Prevention	Australia	2011–2016 (6 years)	Obesity prevention Chronic disease prevention	All ages	Qualitative
6	Bensberg et al., 2021b [44] *Bensberg 2021a and 2021b are related studies: Healthy Together Victoria*	Building a prevention system: Infrastructure to strengthen health promotion outcomes		2012–2016 (5 years)			Qualitative
7	Brimblecombe et al., 2015 [45]	Development of the good food planning tool: A food system approach to food security in indigenous Australian remote communities	Australia	2009–2013 (5 years)	Food security	Indigenous Australian remote communities (all ages)	Mixed methods Case study
8	Buccini et al., 2019 [46]	How does "Becoming Breastfeeding Friendly" work? A Programme Impact Pathways Analysis	Ghana and Mexico	2017–2018 (2 years)	Breastfeeding friendly	Mothers who wish to breastfeed	Mixed methods
9	Gadsby et al., 2020 [47]	Impact of a community-based pilot intervention to tackle childhood obesity: a 'whole-system approach' case study	United Kingdom	2015–2018 (4 years)	Childhood obesity prevention	Children 6-11 years	Mixed methods Case study
11	Kremser, 2011 [48]	Phases of school health promotion implementation through the lens of complexity theory: lessons learnt from an Austrian case study	Austria	2008–2009 (2 years)	Health promoting school	Primary school children (note – primary school is for four years in Vienna)	Mixed methods Case study
12	Matheson et al., 2020 [49]	Strengthening prevention in communities through systems change: lessons from the evaluation of Healthy Families NZ	New Zealand	2014 – 2016 (3 years, intervention ongoing to 2022)	Prevention of chronic diseases	Families	Mixed methods Comparative case study
13	Ramanadhan et al., 2017 [50]	Building practitioner networks to support dissemination and implementation of evidence-based programs in community settings	United States	2010–2012 (3 years)	Adaptation of evidence-based (health) programs in community	All ages	Qualitative Manuscript states: ‘Study design’ The study utilized whole-network (or sociometric) analysis for each of the three communities)
14	Rosas et al., 2019 [51]	Evaluating a complex health promotion intervention: case application of three systems methods	United States	2011–2015 (5 years)	Health and wellbeing of youth	12–15 years	Mixed methods Case study
15	Rothwell et al., 2010 [52]	Implementing a Social-Ecological Model of Health in Wales	United Kingdom	2001- 2007 (7 years)	Healthy schools	Children – primary and secondary age	Qualitative Case study
16	Sautkina et al., 2014 [53]	Lost in translation? Theory, policy and practice in systems-based environmental approaches to obesity prevention in the Healthy Towns programme in England	United Kingdom	2008–2011 (4 years)	Obesity prevention	All ages	Qualitative
17	Serpas et al., 2013 [54]	San Diego Healthy Weight Collaborative: a systems approach to address childhood obesity	United States	2010–2012 (3 years)	Childhood obesity prevention	Children	Mixed methods

The term ‘systems’ was used loosely across the studies (Table 2) which made it difficult to describe what systems approaches looked like in practice. Two studies reported on a multi-component, multi-setting intervention (The Scope Study) grounded on a systems approach [40, 41]. Four explicitly noted the importance of interconnections and relationships within the system under study [43, 44, 54, 55] for example, ….. Related terms were also used loosely. For example, three studies used ‘complexity’ to describe their systems approach [46–48]. Complexity was described as a self-organising system whose ‘whole is not simply a sum of its parts’ (48p.138), or a need to understand the causal mechanisms and impact pathways of policies and programmes [46] or more generally as ‘complex adaptive systems that help to explain particular problematic situations and identify ways in which they might be improved’ ([47], p. 2).

**Table 2:** Definition of systems, type of systems intervention or methods as provided by the authors of included manuscripts and implementation theory framework or model reported

Study ID	System science description, process, model, or method	Implementation Science Theory, framework, model and whether it is a process, determinant or evaluation implementation science framework (in brackets)
Allender et al., 2019 [39]	Community-based participatory system dynamics for the development, implementation, evaluation of whole of community efforts to improve the health of children Group model building	Authors used ‘Essential components of transformative systems change’ (Foster-Fishmann) to provide qualitative insights on implementation and evaluation of implementation (process and evaluation framework)
The SCOPE study Amed et al., 2016 [40] Kennedy et al. 2019 [41]	Complex social problems require 'systems approaches'; multi-component, multi-setting adaptive solutions that are implemented in real-world settings Systems-level/community-level change The manuscript describes its work as: 'a multi-sectoral, multi-component community-based childhood obesity prevention initiative grounded in systems thinking and participatory research principles' p.1 'This type of capacity-building approach grounded in systems thinking and principles of community-based participatory research and collective impact has proven to be an effective response to the complex socio-ecological causes of childhood obesity.' p.2	This study adapted the RE-AIM model with the knowledge translation model described in the study to create RE-FRAME (Reach, engagement, facilitation/coaching/training, resources, adaptation, mobilisation of champions, exchange of knowledge) -(evaluation framework) Knowledge to action (KTA) framework (process framework) Collective impact framework (process framework) RE-FRAME model—based on RE-AIM framework—(evaluation framework)
The ‘Whole School, Whole Community, Whole Child’ study Ballard et al., 2020 [17] Purnell et al., 2020 [42]	Community-based system dynamics (CBSD)—is a participatory approach for engaging communities in understanding and changing complex systems Social network analysis	The Whole School, Whole Community, Whole Child (WSCC) model (process framework)
Healthy Together Victoria study Bensberg 2021a [43] Bensberg et al., 2021b [44]	Systems thinking is a method of problem solving that is concerned with the interconnections between system parts and their relationship to a functioning whole, which cannot be understood by looking at the parts in isolation [58] Complex whole of system approach that required the activation of community-level organizations via multifaceted and interconnected interventions to improve physical inactivity, poor diet, smoking, and harmful alcohol use [59] Systems thinking is an approach for solving complex problems, that emphasises looking at the whole rather than the isolated parts, and highlighting the relationships between the parts, their causal linkages and feedback loops Systems change model (Centre of Excellence in Prevention Science) Strengthening Systems for Chronic Disease Prevention framework (Baugh, Littlejohns and Wilson) A stock and flow diagram A causal loop diagram	Healthy Food Connect implementation guide (Victorian Dept. of Health Human Services) (process framework)
Brimblecombe et al., 2015 [45]	‘A cornerstone of these system approaches is the focus on achieving quality improvement through a structured ongoing cycle of participatory assessment, planning and action, to achieve step- by-step incremental improvement. Through this process of discussion and analysis stakeholders incrementally build their knowledge of the nature of the system and how it behaves.’ p.55	Good Food Planning Tool (author devised tool) (process framework)
Buccini et al., 2019 [46]	‘Becoming Breastfeeding Friendly is grounded in implementation science within the context of complex adaptive systems and the need to understand causal mechanisms and impact pathways of resulting policies and programmes to strengthen its impact and sustainability globally.']p.2	Programme impact pathways analysis (evaluation framework)
Gadsby et al., 2020 [47]	Adopts the Public Health England definition, ‘a local whole systems approach responds to complexity through an ongoing, dynamic and flexible way of working…stakeholders agree actions and decide as a network how to work together in an integrated way to bring about sustainable, long-term systems change’. p.2	RE-AIM framework (evaluation framework)
Kremser, 2011 [48]	Complexity theory underpins the study—systems and subsystems are defined. The classroom level was seen as a system level, distinguished from the school level A systems approach in a school setting	WHO—Health Promoting Schools (WHO_HPS) (process framework)
Matheson et al., 2020 [49]	Healthy Families NZ is a government-funded initiative which takes a systems change approach to strengthening community leadership and organization to prevent chronic disease. It builds on existing action underway in the community to strengthen the health prevention system through evidence-driven action to enable people to make good food choices, be physically active, smoke free and free from alcohol-related harm Described as a systems-change intervention	Five building blocks adapted from WHO Building Blocks for a Strong Health System (process framework) Seven principles adapted from Healthy Together Victoria (process framework)
Ramanadhan et al., 2017 [50]	Defines capacity building as a systems intervention and acknowledges the importance of social relationships for knowledge sharing and practice change Community-based systems approach (capacity building as a systems intervention)	Participatory Approach to Knowledge Translation (PaKT) Framework (process framework)
Rosas et al., 2019 [51]	Defines as per Leischow and Milstein [60] ‘Systems thinking considers connections among different components, anticipates their interaction, embraces transdisciplinary viewpoints, and requires active engagement of stakeholders to govern the course of change.’ p. 338 The manuscript reports an evaluation approach based on systems thinking and complexity science, principles, concepts, and methods	A theory of change was developed (although not reported here), to guide the communities work, and in turn developmental evaluation (GMB, VSM and SNA) was used to guide implementation. (Each community adjusted to their own needs)—(process and evaluation framework)
Rothwell et al., 2010 [52]	‘Systems thinking informs both the Ottawa charter and the social-ecological model of health’ p. 472	Welsh Network of Healthy Schools Scheme national framework based on Ottawa Charter principles (process framework)
Sautkina et al., 2014 [53]	This manuscript refers to the systems definition provided in Butland et al.,’Such a “systems-based” approach would target multiple determinants, at multiple levels throughout the life course [61]. In particular, the report stressed the importance of reshaping the built and social environments in order to facilitate improvements in diet and increase physical activity levels.’	Health promotion teams utilised their own preferred frameworks, no overarching guiding framework was provided (unclear)
Serpas et al., 2013 [54]	A systems approach: ‘explicitly designs intervention strategies to focus on interactions and interconnections (integration) between different sectors in the community, and between the individuals and their environment in that community ‘accounts for the context and characteristics of a community in planning intervention strategies in order to see the whole picture so that intended and unintended consequences of intervention strategies can be recognized and strategies altered if required ‘utilizes a multidisciplinary approach, including community experts, to determine proposed interactions among systems and sectors that will be required to result in feasible interventions that are sustainable (persistence of changes made and ongoing adoption of new ones); scalable (an intervention can be brought to scale to impact many settings); and have reach (across cultural and language population sub-groups).’p. 81	Plan-Do-Study-Act (process framework) Strategy Mapping Exercise (determinants framework)

Consequently, few studies reported using specific systems methods. Three studies reported using community-based system dynamics and group model building [17, 39, 42]. Others were more generic, referring to a systems approach to strengthening community leadership [49], a community-based systems approach with an emphasis on capacity building [50], and a whole of community systems approach [47]. The Scope Study described their multi-component intervention as a ‘modest and early effort’ to incorporate systems approaches within obesity prevention [40, 41]. Contextually, some studies operated at a broad whole of community systems level [39, 47], while others considered the classroom within a school as a ‘system’ [17, 48].

A broad spread of implementation TFMs were used in the studies. Overall included studies used 11 process frameworks, 5 evaluation frameworks and 1 determinants framework (these do not add to 14 because some studies used multiple frameworks). The RE-AIM (reach, effectiveness, adoption, implementation, maintenance) evaluation framework was used in two studies [40, 47]. One of these studies adapted RE-AIM, to include reach, engagement, facilitation, resources, adaptation, mobilisation, and exchange, and re-named to RE-FRAME [40, 41]. One study paired RE-FRAME with the Knowledge-to-Action framework [40, 41]. A different study paired The Plan-Do-Study-Act with a strategy mapping exercise [54]. Other authors used frameworks developed from peak bodies, such as the Health Promoting Schools’ Framework [56], and the Building Blocks for a Strong Health System [57]. One author described a guide for implementation of a systems approach, where local communities developed their own independent methods of implementation [53]. Three of the 14 studies combined two TFMs to guide different stages of implementation. There was no single implementation science framework or set of TFMs favoured to guide the implementation of interventions designed using systems science.

Where authors described the use of both systems and implementation science constructs, these were used in collaboration, systems science was used in the design of the study, implementation science was used to guide real-world implementation of the designed study.

## Discussion

This systematic review identified 14 studies (17 manuscripts) that used a combination of systems thinking approaches and implementation science TFMs to design and deliver health promotion interventions. There was no consistency in the definition of systems science, or the systems methods applied in the studies and a broad spread of TFMs was reported. CBSD was the only systems method used in more than one study [17, 39] and RE-AIM the only TFM used in more than one study, albeit in a substantially modified form in one of these two studies [40, 41, 47].

Braithwaite, in a 2018 opinion piece, stated that “the two sciences of complexity and implementation need not be mutually exclusive, though they have been largely seen and treated as such” ([29], p. 6). Our review confirms this remains the case, and aligns with the call for more systematic reporting of intervention studies that utilise systems approaches [62]. Our findings also support opportunities to build on and strengthen existing theoretical approaches, rather than invent new and untested frameworks [63]. Where Northridge [30] sought to enhance implementation science through the addition of best principles from systems science, our review sought to identify insights from published literature on ways to improve the implementation of systems science approaches to prevention through the incorporation of implementation science TFMs.

A previous review of TFMs used in prevention and/or management of cancer and other chronic diseases classified 159 TFMs against the socio-ecological model (SEM) of health: individual, organisational, community and system [28]. Of these, only 17% (*n* = 27) were deemed to impact the systems level of the SEM, although the term ‘system’ was not defined in the review. Highly cited implementation science TFMs were included such as Social Cognitive Theory [64], Social Leaning Theory [65], Transtheoretical Model of Behaviour Change [66], PRECEDE-PROCEED [67] and Plan-Do-Study-Act [68]. Other TFMs were also included that were less well known or were specifically designed to fit an intervention. Of these, the Plan-Do-Study-Act was the only framework that also appeared in both the SEM review [28] and our review. This limited overlap is likely due to the evolution of systems science, with earlier iterations of the SEM referring to this outer layer as ‘policy’ or ‘social’, rather than ‘systems’. Additionally, most TFMs have historically been applied to either individual behaviour change programs or settings [28], leaving it unclear which TFMs are best suited to a systems approach for community-based prevention intervention.

Many of the included manuscripts did not clearly define ‘systems’ nor clearly articulate the system method used in the study. This observation aligns with a systematic review of whole systems approaches to complex public health problems that found few programs had utilised systems approaches in the study design, implementation and evaluation; and rarely conceptualised implementation from a systems perspective [70]. We concur with Foster-Fishman that systems change requires adopting systems beyond a general definition and adopting a change framework is critical to guide true systems change systemically, rather than within a specific part of the system. For example, there remains tension that ‘policy’ can be misinterpreted as a systems approach, when policy alone may impact only one area of the system and should usually be partnered with other systemic actions [69], such as appropriate resourcing, enabling infrastructure, appropriate skills and knowledge. Without such wrap-around support, a policy is unlikely to have the reach required for a whole of system change. Inadequate framing of ‘systems’ may complicate the choice of appropriate implementation science guidance.

When implementing systems interventions, implementation requires more than just effectiveness planning, but also the anticipation of, and engagement with, a range of contexts, stakeholders and potential consequences [29]. Northridge [30] provided insights from systems science that could enhance implementation science, concepts such as, e.g. problem modelling, the inclusion of important elements rather than quantifiable elements, boundary identification and a multi-component approach. A positive signpost for future integration of the fields of implementation science and systems science is reported modification of TFMs to enhance their applications to complex intervention and systems approaches. In a recent 20-year review of RE-AIM, Glasgow et al. identified one of the future directions for the RE-AIM framework was to incorporate system concepts such as unintended consequences [71]. Such extension of a widely used framework to overtly embrace systems concepts is encouraging. Other wholistic frameworks are constantly evolving to specifically address systems change, such as the Active Implementation Frameworks developed by Fixsen and colleagues [72]. This overarching framework aligns with the interactive nature of systems through its non-linear approach to implementation and inclusion of improvement cycles. These advances in implementation science articulate promise for enhanced guidance for the implementation of systems interventions, aligns with multiple calls to integrate implementation and system science approaches [32, 53], and acknowledges the potential within the field of implementation science as noted in the Lancet’s Commission on Obesity [4].

### Future research

Future studies that utilise systems thinking approaches should more clearly define terminology and specify the systems method employed within the study. Recent innovative trial designs such as stepped wedge designs [73] and hybrid Type II designs [74] may assist in the combination of systems and implementation sciences by enabling efficacy testing of both the systems intervention and the implementation strategies. Future trials, using various combinations of systems thinking and implementation science methods, would assist in identifying a ‘preferred’ combination of these approaches for population health prevention interventions.

Although GMB and RE-AIM were the most identified methods and frameworks within this review, there were too few studies to recommend these methods either alone or in combination to close this gap. Instead, there exists great potential for future research to interrogate the use of clearly defined systems methodologies and implementation science theoretical approaches to enable cyclical implementation. Such a combination has potential to optimise the intrinsic overlap in these two scientific disciplines and identify aligned theories and practice.

### Strengths and limitations

To our knowledge, this is the first systematic review to explore the use of an integrated systems and implementation science approach in public health prevention intervention design and delivery. This review demonstrates there is little practical guidance to date on how best to implement systems approaches for population health. The review included all manuscripts that identified the use of systems—therefore avoiding bias for any preferred systems methodology and including studies where systems thinking was emerging. Similarly, we defined implementation science theoretical approaches broadly to maximise the capture of all manuscripts using any theoretically informed approach to implementation.

Heterogeneity of definitions of both systems science and implementation science means it was difficult to draw conclusions from existing manuscripts as to what the ‘best’ combination of these sciences might be. We limited our review to peer-reviewed literature therefore case studies may exist in the grey literature that were not captured by this review.

The heterogeneity of ‘systems’ terminology means it is possible that some work that draws from principles of systems thinking without using explicit terminology may have not been included in our review. Consequently we were unable to obtain practical guidance on how to apply these dual approaches of systems and implementation sciences.

## Conclusions

To date there is limited alignment between systems thinking and implementation science approaches in the design and delivery of public health prevention interventions. Based on this review, we are unable to recommend the most promising combination of systems thinking methods and implementation science TFMs as the combination of these fields remains underdeveloped. We join the call for consistency of language, definition and guidance on the use and reporting of an integrated systems and implementation science approach for public health prevention interventions.

### Supplementary Information

**Additional file 1:** Search terms.

**Additional file 2:** Details of included studies.

**Additional file 3:** Quality appraisal of included studies.

## Data Availability

All search terms are provided in Additional information, for any further information please contact the corresponding author.

## References

[1] Habib SH, Saha S (2010). Burden of non-communicable disease: global overview. Diabetes Metab Syndr.

[2] Murray CJL, Abbafati C, Abbas KM, Abbasi M, Abbasi-Kangevari M, Abd-Allah F (2020). Five insights from the Global Burden of Disease Study 2019. Lancet.

[3] Brown T, Moore THM, Hooper L, Gao Y, Zayegh A, Ijaz S, et al. Interventions for preventing obesity in children. Cochrane Database Syst Rev. 2019(7). 10.1002/14651858.CD001871.pub4 10.1002/14651858.CD001871.pub4 PMC6646867 31332776

[4] Swinburn BA, Kraak VI, Allender S, Atkins VJ, Baker PI, Bogard JR (2019). The global syndemic of obesity, undernutrition, and climate change: The Lancet Commission report. Lancet.

[5] Bozsik F, Shook R, Wilson E, Carlson J, Markenson D, Meissen-Sebelius E (2021). Characterization of a regional childhood obesity prevention and treatment system. Child Obes.

[6] Sterman J. System dynamics: systems thinking and modeling for a complex world. 2002.

[7] Frank M (2016). Systems thinking: foundation, uses and challenges.

[8] Haines SG (2002). Systems thinking and learning: from chaos and complexity to elegant simplicity. Annual-San Diego-Pfeiffer Company.

[9] Rutter H, Savona N, Glonti K, Bibby J, Cummins S, Finegood DT (2017). The need for a complex systems model of evidence for public health. Lancet.

[10] Savigny Dd, Adam T, Alliance for Health P, Systems R, World Health O. Systems thinking for health systems strengthening / edited by Don de Savigny and Taghreed Adam. Geneva: World Health Organization; 2009.

[11] Stroh DP (2015). Systems thinking for social change : a practical guide to solving complex problems, avoiding unintended consequences, and achieving lasting results.

[12] Bolton KA, Whelan J, Fraser P, Bell C, Allender S, Brown AD (2022). The Public Health 12 framework: interpreting the 'Meadows 12 places to act in a system' for use in public health. Arch Public Health.

[13] Meadows D. Leverage points. Places to intervene in a system. The Sustainability Institute, Hartland Four Corners, Vermont, USA; 1999.

[14] Boelsen-Robinson T, Blake MR, Brown AD, Huse O, Palermo C, George NA (2021). Mapping factors associated with a successful shift towards healthier food retail in community-based organisations: a systems approach. Food Policy.

[15] Carey G, Crammond B (2015). Systems change for the social determinants of health. BMC Public Health.

[16] Allender S, Owen B, Kuhlberg J, Lowe J, Nagorcka-Smith P, Whelan J (2015). A community based systems diagram of obesity causes. PLoS ONE.

[17] Ballard E, Farrell A, Long M (2020). Community-based system dynamics for mobilizing communities to advance school health. J Sch Health.

[18] Gerritsen S, Renker-Darby A, Harre S, Rees D, Raroa DA, Eickstaedt M (2019). Improving low fruit and vegetable intake in children: findings from a system dynamics, community group model building study. PLoS ONE [Electronic Resource].

[19] Cavana RY, Tobias M (2008). Integrative system dynamics: analysis of policy options for tobacco control in New Zealand. Syst Res Behav Sci.

[20] Atkinson J-A, Page A, Heffernan M, McDonnell G, Prodan A, Campos B (2019). The impact of strengthening mental health services to prevent suicidal behaviour. Aust N Z J Psychiatry.

[21] Ison R (2010). Systems practice: how to act in a climate change world.

[22] Luke DA, Stamatakis KA (2012). Systems science methods in public health: dynamics, networks, and agents. Annu Rev Public Health.

[23] Morshed AB, Kasman M, Heuberger B, Hammond RA, Hovmand PS (2019). A systematic review of system dynamics and agent-based obesity models: evaluating obesity as part of the global syndemic. Obes Rev.

[24] Riley B, Willis C, Holmes B, Finegood D, Best A, McIsaac J. Systems thinking and dissemination and implementation research. Dissemination and implementation research in health. Transl Sci Pract. 2017;143.

[25] Lobb R, Colditz GA (2013). Implementation science and its application to population health. Annu Rev Public Health.

[26] Nilsen P (2020). Making sense of implementation theories, models, and frameworks. Implement Sci.

[27] Swinburn B, Dietz W, Kleinert S (2015). A Lancet Commission on obesity. Lancet.

[28] Strifler L, Cardoso R, McGowan J, Cogo E, Nincic V, Khan PA (2018). Scoping review identifies significant number of knowledge translation theories, models, and frameworks with limited use. J Clin Epidemiol.

[29] Braithwaite J, Churruca K, Long JC, Ellis LA, Herkes J (2018). When complexity science meets implementation science: a theoretical and empirical analysis of systems change. BMC Med.

[30] Northridge ME, Metcalf SS (2016). Enhancing implementation science by applying best principles of systems science. Health Res Policy Syst.

[31] Hovmand P (2014). Community based system dynamics.

[32] Gerritsen S, Harre S, Rees D, Renker-Darby A, Bartos AE, Waterlander WE (2020). Community group model building as a method for engaging participants and mobilising action in public health. Int J Environ Res Public Health [Electronic Resource].

[33] Kania A, Patel AB, Roy A, Yelland GS, Nguyen DTK, Verhoef MJ (2012). Capturing the complexity of evaluations of health promotion interventions: a scoping review. Can J Program Eval.

[34] Hennessy E, Economos CD, Hammond RA (2020). Integrating complex systems methods to advance obesity prevention intervention research. Health Educ Behav.

[35] Moher D, Liberati A, Tetzlaff J, Altman DG, Group P (2009). Preferred reporting items for systematic reviews and meta-analyses: the PRISMA statement. Ann Intern Med.

[36] Malhi L, Karanfil O, Merth T, Acheson M, Palmer A, Finegood DT (2009). Places to intervene to make complex food systems more healthy, green, fair, and affordable. J Hunger Environ Nutr.

[37] Hong QN, Fàbregues S, Bartlett G, Boardman F, Cargo M, Dagenais P (2018). The Mixed Methods Appraisal Tool (MMAT) version 2018 for information professionals and researchers. Educ Inf.

[38] Page MJ, McKenzie JE, Bossuyt PM, Boutron I, Hoffmann TC, Mulrow CD (2021). The PRISMA 2020 statement: an updated guideline for reporting systematic reviews. Syst Rev.

[39] Allender S, Brown AD, Bolton KA, Fraser P, Lowe J, Hovmand P (2019). Translating systems thinking into practice for community action on childhood obesity. Obes Rev.

[40] Amed S, Shea S, Pinkney S, Higgins JW, Naylor PJ (2016). Wayfinding the live 5-2-1-0 initiative—at the intersection between systems thinking and community-based childhood obesity prevention. Int J Environ Res Public Health.

[41] Kennedy L, Pinkney S, Suleman S, Mâsse LC, Naylor P-J, Amed S (2019). Propagating change: using RE-FRAME to scale and sustain a community-based childhood obesity prevention initiative. Int J Environ Res Public Health.

[42] Purnell JQ, Lobb Dougherty N, Kryzer EK, Bajracharya S, Chaitan VL, Combs T (2020). Research to translation: the healthy schools toolkit and new approaches to the whole school, whole community, whole child model. J Sch Health.

[43] Bensberg M (2021). Developing a systems mindset in community-based prevention. Health Promot Pract.

[44] Bensberg M, Allender S, Sacks G (2020). Building a systems thinking prevention workforce. Health Promot J Austr.

[45] Brimblecombe J, Van Den Boogaard C, Wood B, Liberato SC, Brown J, Barnes A (2015). Development of the good food planning tool: a food system approach to food security in Indigenous Australian remote communities. Health Place.

[46] Buccini G, Harding KL, Hromi-Fiedler A, Perez-Escamilla R (2019). How does "Becoming Breastfeeding Friendly" work? A programme impact pathways analysis. Matern Child Nutr.

[47] Gadsby EW, Hotham S, Eida T, Lawrence C, Merritt R (2020). Impact of a community-based pilot intervention to tackle childhood obesity: a 'whole-system approach' case study. BMC Public Health.

[48] Kremser W (2011). Phases of school health promotion implementation through the lens of complexity theory: lessons learnt from an Austrian case study. Health Promot Int.

[49] Matheson A, Walton M, Gray R, Wehipeihana N, Wistow J (2020). Strengthening prevention in communities through systems change: lessons from the evaluation of Healthy Families NZ. Health Promot Int.

[50] Ramanadhan S, Minsky S, Martinez-Dominguez V, Viswanath K (2017). Building practitioner networks to support dissemination and implementation of evidence-based programs in community settings. Transl Behav Med.

[51] Rosas S, Knight E (2019). Evaluating a complex health promotion intervention: case application of three systems methods. Crit Public Health.

[52] Rothwell H, Shepherd M, Murphy S, Burgess S, Townsend N, Pimm C (2010). Implementing a social-ecological model of health in Wales. Health Educ.

[53] Sautkina E, Goodwin D, Jones A, Ogilvie D, Petticrew M, White M (2014). Lost in translation? Theory, policy and practice in systems-based environmental approaches to obesity prevention in the Healthy Towns programme in England. Health Place.

[54] Serpas S, Brandstein K, McKennett M, Hillidge S, Zive M, Nader PR (2013). San Diego Healthy Weight Collaborative: a systems approach to address childhood obesity. J Health Care Poor Underserved.

[55] Rosas SR (2017). Systems thinking and complexity: considerations for health promoting schools. Health Promot Int.

[56] Langford R, Bonell C, Jones H, Pouliou T, Murphy S, Waters E (2015). The World Health Organization’s Health Promoting Schools framework: a Cochrane systematic review and meta-analysis. BMC Public Health.

[57] Organization WH. Monitoring the building blocks of health systems: a handbook of indicators and their measurement strategies. World Health Organization; 2010.

[58] Mabry PL, Olster DH, Morgan GD, Abrams DB (2008). Interdisciplinarity and systems science to improve population health: a view from the NIH Office of Behavioral and Social Sciences Research. Am J Prev Med.

[59] Strugnell C, Millar L, Churchill A, Jacka F, Bell C, Malakellis M (2016). Healthy together Victoria and childhood obesity—a methodology for measuring changes in childhood obesity in response to a community-based, whole of system cluster randomized control trial. Arch Public Health.

[60] Leischow SJ, Milstein B. Systems thinking and modeling for public health practice. American Public Health Association; 2006. p. 403–5. 10.2105/AJPH.2005.082842 PMC1470500 16449572

[61] Butland B, Jebb S, Kopelman P, McPherson K, Thomas S, Mardell J, et al. Tackling obesities: future choices-project report: Citeseer; 2007. 10.1111/j.1467-789X.2007.00344.x 17316292

[62] Li B, Allender S, Swinburn B, Alharbi M, Foster C. Improving the reporting of intervention studies underpinned by a systems approach to address obesity or other public health challenges. Front Public Health. 2022;10. 10.3389/fpubh.2022.892931 PMC9360742 35958870

[63] Wensing M, Grol R (2019). Knowledge translation in health: how implementation science could contribute more. BMC Med.

[64] Bandura A. Social-cognitive theory. An introduction to theories of personality. 2014:341–60.

[65] Bandura A, Walters RH (1977). Social learning theory.

[66] Sutton S, French D, Hennings S (1997). Transtheoretical model of behaviour change. Curr Psychol.

[67] Gielen AC, McDonald EM, Gary TL, Bone LR (2008). Using the precede-proceed model to apply health behavior theories. Health Behav Health Educ Theory, Res Pract.

[68] Taylor MJ, McNicholas C, Nicolay C, Darzi A, Bell D, Reed JE (2014). Systematic review of the application of the plan–do–study–act method to improve quality in healthcare. BMJ Qual Saf.

[69] Foster-Fishman PG, Nowell B, Yang H (2007). Putting the system back into systems change: a framework for understanding and changing organizational and community systems. Am J Community Psychol.

[70] Bagnall A-M, Radley D, Jones R, Gately P, Nobles J, Van Dijk M (2019). Whole systems approaches to obesity and other complex public health challenges: a systematic review. BMC Public Health.

[71] Glasgow RE, Harden SM, Gaglio B, Rabin B, Smith ML, Porter GC (2019). RE-AIM planning and evaluation framework: adapting to new science and practice with a 20-year review. Front Public Health.

[72] Fixsen DL, Blase KA (2020). Active implementation frameworks. Handbook on implementation science.

[73] Brown CA, Lilford RJ (2006). The stepped wedge trial design: a systematic review. BMC Med Res Methodol.

[74] Curran GM, Bauer M, Mittman B, Pyne JM, Stetler C (2012). Effectiveness-implementation hybrid designs: combining elements of clinical effectiveness and implementation research to enhance public health impact. Med Care.

